# Metabolic Engineering of *Escherichia coli* for Poly(3-hydroxybutyrate) Production under Microaerobic Condition

**DOI:** 10.1155/2015/789315

**Published:** 2015-04-07

**Authors:** Xiao-Xing Wei, Wei-Tao Zheng, Xue Hou, Jian Liang, Zheng-Jun Li

**Affiliations:** ^1^Department of Basic Medicine, Medical College of Qinghai University, Xining 810016, China; ^2^Beijing Key Laboratory of Bioprocess, College of Life Science and Technology, Beijing University of Chemical Technology, Beijing 100029, China; ^3^College of Eco-Environmental Engineering of Qinghai University, Xining 810016, China

## Abstract

The alcohol dehydrogenase promoter P_adhE_ and mixed acid fermentation pathway deficient mutants of *Escherichia coli* were employed to produce poly(3-hydroxybutyrate) (P3HB) under microaerobic condition. The *E. coli* mutant with *ackA-pta, poxB, ldhA*, and *adhE* deletions accumulated 0.67 g/L P3HB, up to 78.84% of cell dry weight in tube cultivation. The deletion of pyruvate formate-lyase gene *pflB* drastically decreased P3HB production and P3HB content to 0.09 g/L and 24.44%, respectively. Overexpressing *pflB* via the plasmid in its knocked out mutant restored cell growth and P3HB accumulation, indicating the importance of the pyruvate formate-lyase in microaerobic carbon metabolism. The engineered *E. coli* BWapld (pWYC09) produced 5.00 g/L P3HB from 16.50 g/L glucose in 24 h batch fermentation, and P3HB production yield from glucose was 0.30 g/g, which reached up to 63% of maximal theoretical yield.

## 1. Introduction

Poly(3-hydroxybutyrate) (P3HB) is a polyester of 3-hydroxybutyrate that is synthesized by a variety of microorganisms as intracellular carbon and energy storages under unbalanced nutrients conditions [1]. The mechanical properties of P3HB are similar to those of petrol derived polymers such as polypropylene, which makes it a promising substitution of common plastics [2]. Moreover, P3HB can be obtained from renewable carbon sources and easily degraded into CO_2_ and H_2_O when discarded in natural environments. Thus, microbial production of P3HB via fermentation has attracted extensive attentions during the past few decades [3].

Although considerable efforts have been devoted to decreasing the production cost of P3HB to make it economically competitive, the present price of P3HB is still not feasible to replace traditional petro-based plastics. Generally, P3HB was mainly produced from renewable carbon sources through aerobic fermentation [4, 5]. Given that the feedstock especially carbon source accounts up to a large portion of the production cost, low cost substrates such as crude glycerol and agricultural wastes have been explored for P3HB production [4, 6, 7]. On the other hand, aerobic fermentation process can be high demand of stir and air supply and contribute further energy cost besides feedstock. Microaerobic process could alleviate the energy requirement for fermentation by simplifying the bioreactor design and control strategies. Considering the potential advantages, P3HB production under microaerobic conditions has been investigated [8]. However, the P3HB content obtained under fed-batch microaerobic cultures was 51%, lower than that produced by aerobic fermentation [9]. Low polymer content decreases the purification efficiency and results in extra expenses in downstream process.

To achieve high P3HB content under microaerobic condition, the native promoter of P3HB biosynthesis operon* phaCAB* from* Ralstonia eutropha* was changed to the anaerobic alcohol dehydrogenase promoter P_adhE_, which was reported to be upregulated under oxygen limited condition. As a result, P3HB content was improved from 30% to 48% of cell dry weight [10]. Moreover,* E. coli* undertakes mixed acid fermentation pathway, producing lactate, succinate, acetate, formate, and ethanol under oxygen limited conditions, which decreases the carbon flux into P3HB accumulation.* E. coli* mutant with deletions of* ackA*-*pta*,* poxB*,* ldhA*, and* adhE* was constructed and doubled the cell dry weight and improved P3HB production by 3.5-folds compared to the control [11].

**Figure 1:**
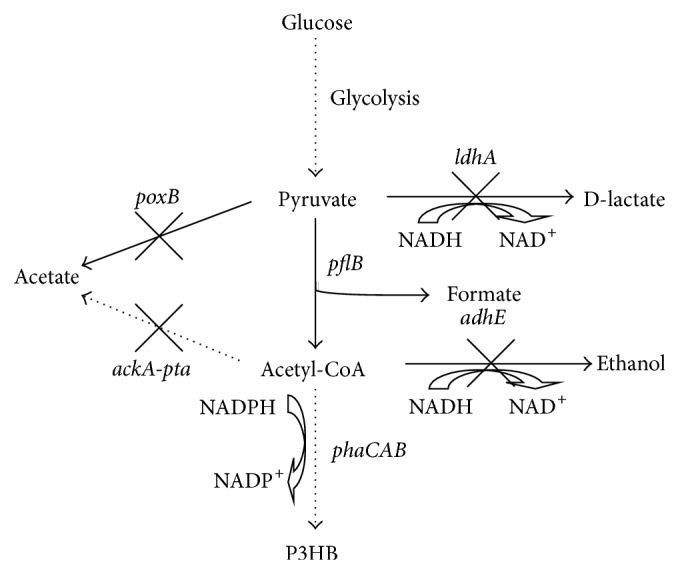
Strategies for improving P3HB production under microaerobic condition in* E. coli.*

In this paper, we managed to further increase the microaerobic P3HB production by employing the P_adhE_ promoter to drive the expression of* phaCAB* operon in* E. coli* mutants with defected mixed acid fermentation pathway. Moreover, the pyruvate formate-lyase, which catalyzes the coenzyme A-dependent, nonoxidative cleavage of pyruvate to acetyl-CoA and formate under anaerobic or microaerobic conditions, was overexpressed to investigate its effect on cell growth and P3HB accumulation (Figure 1).

## 2. Materials and Methods

### 2.1. Bacterial Strains and Plasmids Construction

Bacterial strains, plasmids, and primers used in this study were listed in Table 1.* E. coli* BW25113 mutants with serial deletions of* ackA*-*pta*,* poxB*,* ldhA*,* adhE*, and* pflB* were constructed to eliminate the mixed acid fermentation pathway. Plasmid pWYC09 was constructed to express* phaCAB* operon from* R. eutropha* under the control of P_adhE_. The pyruvate decarboxylase promoter of* Zymomonas mobilis* was amplified with primers pdcF/pdcR and then ligated into the* Xho*I/*Nde*I site of pBBR1MCS-2 to construct pMCS2pdc.* pflB* was amplified from* E. coli* BW25113 genome with primers pflF/pflR and inserted into the* Nde*I/*Eco*RI site of pMCS2pdc to generate pMCS2pflB.

### 2.2. Culture Conditions

For tube cultivation, one percent seed culture was inoculated into a 250 mL sealed tube completely filled with Luria-Bertani (LB) medium supplemented with 10 g/L glucose and then maintained at 37°C for 48 h as static cultures. For 5.5 liter bioreactor cultivation, 50 mL seed culture was transferred to the bioreactor containing 3 L LB medium supplemented with 20 g/L glucose. The pH was maintained at 7.0 via automatic addition of 5 M sodium hydroxide solution. Batch cultures were performed without air supply; the agitation was set at 75 rpm to prevent biomass sedimentation and maintain heat transfer and substrate exchange.

### 2.3. Analytical Methods

Cells were harvested by centrifugation at 8,000 ×g and 4°C for 10 min. The cell pellets were washed twice with distilled water and then lyophilized for 12 h for cell dry weight (CDW) assay. Lyophilized cells were subjected to methanolysis at 100°C for 4 h in the presence of 3% (v/v) H_2_SO_4_ and then assayed with a gas chromatograph (GC) to measure the P3HB content. For glucose and by-products lactate, succinate, acetate, formate, and ethanol measurements, the supernatant of cell cultures was filtered through a 0.2 *μ*m syringe filter and then analyzed by high-performance liquid chromatography (HPLC) equipped with an ion exchange column (Aminex HPX-87H, 300 × 7.8 mm) and a refractive index detector.

## 3. Results and Discussion

### 3.1. P_adhE_ Controlled P3HB Production in* E. coli* Mixed Acid Fermentation Mutants

The alcohol dehydrogenase promoter P_adhE_ has been demonstrated to improve the expression level of* phaCAB* operon under microaerobic condition [10]. Moreover, the disruption of mixed acid fermentation pathway was proved to be another effective strategy to increase P3HB accumulation [11]. To investigate the possibility of the synergistic effect, plasmid pWYC09 carrying P_adhE_ controlled* phaCAB* was transformed into the wild type* E. coli* and a series of mixed acid fermentation mutants BWa, BWap, BWapl, BWapld, and BWapldf (Table 1). The recombinant strains were cultivated in LB medium supplemented with glucose to produce P3HB under microaerobic tubes. The mixed acid fermentation mutants showed higher P3HB content compared to that of wild type except for the* pflB* mutant, and P3HB accumulated up to 78.84% of cell dry weight in the quadruple* ΔackA*-*pta*,* ΔpoxB*,* ΔldhA*, and* ΔadhE* deletion mutant BWapld, which produced the fewest amount of mixed acid (Table 2). Compared to the strains containing* phaCAB* operon with its native promoter, P_adhE_ driven operon led to much higher P3HB production [11].

When* pflB* was knocked out, both cell growth and P3HB accumulation decreased significantly (Table 2). It was reported that the expression of pyruvate formate-lyase was induced to generate acetyl-CoA and formate from pyruvate when* E. coli* was cultivated with limited oxygen. Therefore, the deletion of* pflB* may cause the disruption of glucose metabolism and led to much lower cell biomass and P3HB accumulation. The profile of by-product formation was similar to the previous report [11]. Lactate production was totally eliminated by deleting* ldhA*, and only minute amount of acetate and succinate was detected in BWapld and BWapldf (Table 2).

### 3.2. Effect of* pflB* Overexpression on Cell Growth, P3HB Accumulation, and By-Product Formation

Considering the importance of* plfB* in glucose metabolism under oxygen limited condition, plasmid pMCS2pflB was constructed to constitutively expressing* pflB* and cotransformed with pWYC09 into* E. coli* to study its influence on cell metabolism profile. The plasmid carrying* pflB* overexpression restored the cell growth and P3HB production completely in* pflB* deletion mutant BWapldf. Cell dry weight was increased from 0.36 g/L to 0.70 g/L, and P3HB content was increased from 24.44% to 44.57%, which confirmed the crucial role of* pflB* during the microaerobic P3HB production (Table 3). However,* pflB* overexpression did not affect the cell growth or P3HB accumulation significantly in other mutants; only a slight improvement in terms of the CDW and P3HB content was observed in BWapld mutant. Compared to those of the control without* pflB* overexpression, BWapld harboring pWYC09 and pMCS2pflB grew up to 1.16 g/L CDW and accumulated 84.79% of P3HB, exhibiting 37% and 8% increase, respectively (Table 3). These results indicate that the expression of* pflB* from its native genomic copy could satisfy the requirement of* E. coli* cells for most of cases of experimental condition, and its expression was proved be of crucial importance to oxygen limited glucose metabolism. Furthermore, no large amount of formate production was observed in all* pflB* overexpressing strains, probably due to the expression of formate dehydrogenase, converting formate to CO_2_ and H_2_O when formate amount exceeds the tolerance of* E. coli* [12].

### 3.3. Growth and P3HB Production by* E. coli* BWapld Mutants in a 5.5-Liter Fermenter

**Figure 2:**
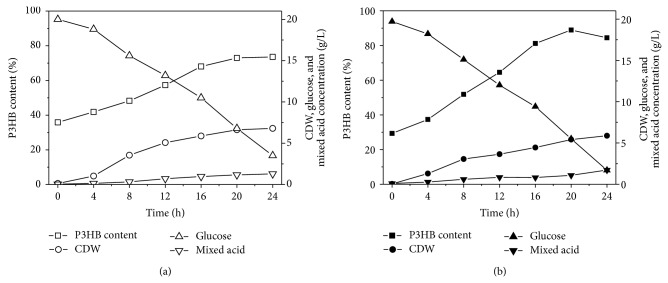
Cell dry weight (CDW), P3HB content (%), glucose consumption, and mixed acid production profile of* E. coli* BWapld harboring P_adhE_ controlled* phaCAB* operon without (a) or with (b)* pflB* overexpression in 5.5 liter bioreactor under microaerobic condition.

The recombinant* E. coli* BWapld (pWYC09) and BWapld (pWYC09 + pMCS2pflB) were cultivated in oxygen limited 5.5 liter bioreactor to investigate cell growth and P3HB production profile. After 24 h cultivation, the recombinant BWapld (pWYC09) consumed 16.50 g/L glucose and produced 5.00 g/L P3HB; the P3HB content reached 73.58% of cell dry weight. The by-product formation was maintained at low level during the process, and finally 0.23 g/L succinate, 0.86 g/L acetate, 0.17 g/L formate, and 0.03 g/L ethanol were detected (Figure 2(a)). When* pflB* was overexpressed, the final CDW and P3HB content were 5.88 g/L and 84.43%, respectively, accompanied with a total amount of 1.72 g/L mixed acid (Figure 2(b)). Previously, the wild type strain BW25113 harboring the same plasmid produced 6.72 g/L CDW with 44.53% P3HB under the same condition. In this study, with the disruption of mixed acid fermentation pathways, the recombinant accumulated P3HB up to 73.58% of cell dry weight, and overexpressing* pflB* helped to further improve P3HB content to 84.43%, which will facilitate downstream biopolymer purification processes.

In terms of byproduct formation and P3HB production yield, the wild type strain BW25113 produced 13.60 g/L mixed acids, and* pta* mutant JW2294 generated 16.60 g/L mixed acids (Table 4). In this study, the mixed acid fermentation pathway mutant BWapld only produced 1.29 g/L mixed acids. As a result, the P3HB production yield from glucose was significantly increased. The wild type obtained 0.10 g P3HB/g glucose, and the* pta* mutant showed a 70% increase to 0.17 g/g. BWapld mutant reached up to 0.30 g/g, which was 2 times higher than that obtained from the wild type (Table 4). The maximal theoretical yield of P3HB from glucose is 0.48 g P3HB/g glucose. Previously, the* arcA* mutant showed 46% of maximal yield under optimized microaerobic fermentation condition [9]. The recombinant constructed in this study reached up to 63% of maximal yield. Hence, the engineered* E. coli* constructed here showed superior ability for microaerobic P3HB production compared to previous strains. Further optimization of different degrees of oxygen limitation in bioreactor batch cultivations should be performed to obtain much higher P3HB production titer and productivity. Since the overall redox balance is important to P3HB biosynthesis and the metabolic response [13], P3HB production with other substrates with different oxidation states of the carbon atoms such as gluconate and glycerol could be another choice for improvements under microaerobic condition.

## 4. Conclusion

The alcohol dehydrogenase promoter and mixed acid fermentation mutants were combined to improve P3HB production under microaerobic condition. The deletion of* pflB* gene significantly decreased cell biomass and P3HB production and overexpressing* pflB* from the plasmid restored the cell growth and P3HB accumulation, indicating the importance of pyruvate formate-lyase for microaerobic metabolism. The engineered strain BWapld harboring pWYC09 accumulated 5.00 g/L P3HB, up to 73.58% of cell dry weight in 5.5 liter bioreactor fermentation. The P3HB production yield from glucose was 0.30 g/g, up to 63% of maximal theoretical yield. The optimization of* phaCAB* expression in mixed acid fermentation mutants was proved to be effective for improving microaerobically P3HB production.

## Figures and Tables

**Table 1:** Strains, plasmids, and primers used in this study.

	Description	Reference

*E. coli* strains		
BW25113	*lacIq rrnBT*14* ΔlacZWJ*16* hsdR*514* ΔaraBADAH*33* ΔrhaBADLD*78	[11]
BWa	BW25113 *ΔackA*-*pta *	[11]
BWap	BW25113 *ΔackA*-*pta*,* ΔpoxB *	[11]
BWapl	BW25113 *ΔackA*-*pta*,* ΔpoxB*,* ΔldhA *	[11]
BWapld	BW25113 *ΔackA*-*pta*,* ΔpoxB*,* ΔldhA*, *ΔadhE *	[11]
BWapldf	BW25113 *ΔackA*-*pta*,* ΔpoxB*,* ΔldhA*, *ΔadhE*, *ΔpflB *	[11]
Plasmids		
pWYC09	pBluescript II SK^−^ derivatives containing* phaCAB* from *R. eutropha* with promoter P_adhE_, Amp^R^	[10]
pBBR1MCS-2	Broad-host-range plasmid, Kan^R^	[10]
pMCS2pdc	Promoter P_pdc_ inserted into pBBR1MCS-2	This study
pMCS2pflB	*pflB* inserted into pMCS2pdc	This study
Primers		
pdcF	5′-ATACTCGAGTTACGCTCATGATCGCGGCATGTC	
pdcR	5′-CCCCATATGTTACTCCATATATTCAAAAC	
plfF	5′-GCTAGGCATATGTCCGAGCTTAATGAAAA	
plfR	5′-CCGAATTCTTACATAGATTGAGTGAAGGT	

All oligonucleotides were synthesized by AuGCT Biotechnology (Beijing, China). Restriction endonuclease digestion sites were underlined.

**Table 2:** P3HB accumulation and by-product formation of *E. coli* BW25113 and its five mutants harboring P_adhE_ controlled *phaCAB* operon.

*E. coli *	CDW (g/L)	P3HB content (%)	Glucose consumed (g/L)	Succinate (g/L)	Lactate (g/L)	Formate (g/L)	Acetate (g/L)	Ethanol (g/L)

BW25113	1.24 ± 0.08	44.85 ± 1.46	7.39 ± 0.09	0.34 ± 0.04	1.21 ± 0.12	0.06 ± 0.01	1.59 ± 0.08	0.25 ± 0.04
BWa	0.73 ± 0.10	68.31 ± 4.86	5.31 ± 0.16	0.37 ± 0.06	1.47 ± 0.26	0.04 ± 0.01	0.35 ± 0.02	0.19 ± 0.02
BWap	0.57 ± 0.06	70.69 ± 5.35	4.89 ± 0.24	0.28 ± 0.06	1.41 ± 0.16	0.03 ± 0.01	0.26 ± 0.06	0.21 ± 0.04
BWapl	1.33 ± 0.08	73.58 ± 4.72	6.26 ± 0.18	0.58 ± 0.08	ND	0.06 ± 0.02	0.45 ± 0.04	0.53 ± 0.06
BWapld	0.85 ± 0.04	78.84 ± 3.27	5.14 ± 0.23	0.32 ± 0.04	ND	ND	0.31 ± 0.02	ND
BWapldf	0.36 ± 0.03	24.44 ± 3.95	2.23 ± 0.08	0.18 ± 0.02	ND	ND	0.20 ± 0.05	ND

The recombinants harboring pWYC09 were cultivated in 250 mL sealed tubes completely filled with LB medium supplemented with 10 g/L glucose at 37°C for 48 h. CDW: cell dry weight. ND: not detected. Data shown are the average and standard deviation of three parallel experiments.

**Table 3:** Influence of *pflB* overexpression on cell growth, P3HB accumulation, and by-product formation.

*E. coli *	CDW (g/L)	P3HB content (%)	Glucose consumed (g/L)	Succinate (g/L)	Lactate (g/L)	Formate (g/L)	Acetate (g/L)	Ethanol (g/L)
BW25113	0.85 ± 0.03	38.44 ± 5.91	6.95 ± 0.17	0.68 ± 0.03	0.97 ± 0.09	0.23 ± 0.02	1.68 ± 0.01	0.45 ± 0.06
BWa	0.58 ± 0.08	71.58 ± 6.93	5.31 ± 0.13	0.72 ± 0.06	1.29 ± 0.12	0.20 ± 0.01	0.64 ± 0.06	0.29 ± 0.04
BWap	0.64 ± 0.02	66.22 ± 4.67	4.89 ± 0.12	0.64 ± 0.01	1.16 ± 0.08	0.18 ± 0.02	0.24 ± 0.07	0.25 ± 0.03
BWapl	1.12 ± 0.07	76.47 ± 2.98	5.36 ± 0.26	0.84 ± 0.02	ND	0.11 ± 0.04	0.29 ± 0.04	0.38 ± 0.05
BWapld	1.16 ± 0.05	84.79 ± 2.37	6.24 ± 0.32	0.85 ± 0.05	ND	0.25 ± 0.06	0.35 ± 0.04	ND
BWapldf	0.70 ± 0.06	44.57 ± 4.02	3.78 ± 0.26	0.73 ± 0.02	ND	0.15 ± 0.04	0.16 ± 0.05	ND

The recombinants harboring pWYC09 and pMCS2pflB were cultivated in 250 mL sealed tubes completely filled with LB medium supplemented with 10 g/L glucose at 37°C for 48 h. CDW: cell dry weight. ND: not detected. Data shown are the average and standard deviation of three parallel experiments.

**Table 4:** Summary of engineered *E. coli *for improving P3HB production under microaerobic condition.

*E. coli *	Description	Cell dry weight (g/L)	P3HB content (%)	P3HB concentration (g/L)	Mixed acid (g/L)	Yield (g P3HB/g glucose)

BW25113	Wild type	6.72	44.53	3.06	13.60	0.10

JW2294	*pta* knockout	7.75	64.31	4.98	16.60	0.17

BWapld	Mixed acid fermentation deficient	6.79	73.58	5.00	1.29	0.30

BWapld (pMCS2pflB)	Mixed acid fermentation deficient and *pflB* overexpression	5.88	84.43	4.96	1.72	0.27

All* E. coli* strains carried plasmid pWYC09, which harbored *phaCAB* operon under the control of P_adhE_. Mixed acid referred the total amount of succinate, formate, acetate, and ethanol.
